# Response Surface Methodology (RSM) on the Hybrid Nanofluid Flow Subject to a Vertical and Permeable Wedge

**DOI:** 10.3390/nano12224016

**Published:** 2022-11-15

**Authors:** Najiyah Safwa Khashi’ie, Iskandar Waini, Mohd Fariduddin Mukhtar, Nurul Amira Zainal, Khairum Bin Hamzah, Norihan Md Arifin, Ioan Pop

**Affiliations:** 1 Fakulti Teknologi Kejuruteraan Mekanikal dan Pembuatan, Universiti Teknikal Malaysia Melaka, Hang Tuah Jaya, Durian Tunggal 76100, Melaka, Malaysia; 2 Institute for Mathematical Research, Universiti Putra Malaysia (UPM), Serdang 43400, Selangor, Malaysia; 3 Department of Mathematics, Faculty of Science, Universiti Putra Malaysia (UPM), Serdang 43400, Selangor, Malaysia; 4 Department of Mathematics, Babeş-Bolyai University, 400084 Cluj-Napoca, Romania

**Keywords:** dual solutions, experimental design, heat transfer, hybrid nanofluid, mixed convection, suction

## Abstract

The mixed convection flow with thermal characteristics of a water-based Cu-Al_2_O_3_ hybrid nanofluid towards a vertical and permeable wedge was numerically and statistically analyzed in this study. The governing model was constructed using physical and theoretical assumptions, which were then reduced to a set of ordinary differential equations (ODEs) using similarity transformation. The steady flow solutions were computed using the Matlab software bvp4c. All possible solutions were presented in the graphs of skin friction coefficient and thermal rate. The numerical results show that the flow and thermal progresses are developed by enhancing the controlling parameters (wedge parameter, volumetric concentration of nanoparticles, and suction parameter). Moreover, the response surface methodology (RSM) with analysis of variance (ANOVA) was employed for the statistical evaluation and conducted using the fit general linear model in the Minitab software. From the standpoint of statistical analysis, the wedge parameter and volumetric nanoparticle concentration have a considerable impact on all responses; however, the suction parameter effect is only substantial for a single response.

## 1. Introduction

The utilization of nanofluid as a conductive coolant is one of the most well-known methods that ensure great thermal performance at a low cost. Nanofluid is formed by the homogeneous combination of extremely small nanoscale particles and a base fluid. Shah et al. [1] analyzed the mass transport and hydro-thermal characteristics with the convective flow of a non-Newtonian micropolar fluid with copper oxide nanomaterial and a mixture of pure water and ethylene glycol subjected to an electromagnetic surface. They found that the micropolarity and electrical conducting of the nanofluidic medium play an important role in the nanofluid motion. Moreover, the flow and thermal characteristics of alumina–water nanofluid with different nanoparticle shapes (sphere, platelet, cylinder, and brick) due to a rotating disk were scrutinized by Sabu et al. [2]. They concluded that the highest drag was contributed by the platelet-shaped alumina, followed by the cylinder-, brick-, and sphere-shaped alumina. Another numerical study regarding the nanofluid flow subjected to the Riga surface in a Darcy–Forchheimer porous medium was conducted by Rasool et al. [3]. They revealed that a significant enhancement in the thermal rate could be achieved by manipulating the electromagnetic planar support and convective heating process. Hybrid nanofluids are the latest generation of heat transfer fluids that offer high heat transfer compared to conventional fluids as the hybrid nanoparticles increase the thermal conductivity of the fluids. Jana et al. [4] experimentally investigated the enhancement of thermal conductivity in hybrid nanoparticles. Suresh et al. [5] reported the advantage of hybrid nanoparticles in the enhancement of the fluid thermal conductivity, which was then continued by Takabi and Salehi [6] and Devi and Devi [7]. Several studies on hybrid nanofluid incorporated with the non-Newtonian fluid model were reported by researchers [8,9,10,11]. Nabwey and Mahdy [8] considered the micropolar hybrid nanofluid flow through a porous medium with dusty particles. Similarly, Roy et al. [9] investigated the flow of micropolar hybrid nanofluid over a shrinking sheet. The thermal performance of peristaltic flow utilizing the hybrid nanoparticles in Eyring–Powell fluid was reported by Riaz et al. [10]. Additionally, Khashi’ie et al. [11] considered the stagnation point flow of second-grade fluid containing hybrid nanoparticles towards a Riga plate. The hybrid nanofluid flow over the Riga channel with slip conditions was studied by Abbas et al. [12], while Waqas et al. [13] and Bilal et al. [14] considered the rotating disk and inclined cylinder geometries, respectively. Uysal and Korkmaz [15] and Kumar and Sarkar [16] considered the hybrid nanofluid flow in a mini channel. Further discussions regarding the thermal and flow characteristics of hybrid nanofluids were reported by Salehi et al. [17], Zainal et al. [18,19,20], Khashi’ie et al. [21,22,23,24], Waini et al. [25,26,27,28,29] and Shah et al. [30].

In recent decades, the flow through a wedge-shaped surface has gained much attention due to its extensive uses in the engineering and chemical industry, such as in the fields of geothermal energy and aerodynamics. The pioneering study in wedge flow was initiated by Falkner and Skan [31] and is known as Falkner–Skan flow. Later, the pressure gradient was considered in this model by Hartree [32] and called a Hartree pressure gradient parameter. Since then, the wedge flow with the various effect of physical parameters has been published, see Refs. [33,34,35,36,37,38,39]. Moreover, the moving wedge flow was examined by Ishak et al. [40,41,42], Khan and Pop [43], and Hedayati et al. [44]; additionally, the shrinking wedge surface was reported by Alam et al. [45], Khan et al. [46], Awaludin et al. [47], and Waini et al. [48]. In recent years, the effect of the nanoparticles on wedge flow has been reported by researchers, for example, Rashad [49], Hassan et al. [50], Ahmed et al. [51], Zaib and Haq [52], Rawat et al. [53], and Mahanthesh et al. [54].

There are numerous advantages to employing the design of experiment (DOE) in research with various factors and outcomes. Response surface methodology (RSM) is one of the design types. RSM is a statistical method widely used for modeling and analyzing processes in which the response of interest is affected by multiple variables, where the goal of the method is to maximize the response [55,56]. The primary advantage of the RSM is that it saves time and money by reducing the number of trials required. The RSM can be summarized as a method for determining how independent variables interact. Based on the dataset, an analysis of variance (ANOVA) was performed to determine whether or not the variables in the experiment were statistically significant. The application of RSM, including the ANOVA, was discussed by Mehmood et al. [57] for the rotating disk flow problem. There are also many fluid flow problems that have been reported with the RSM and statistical data analysis (see Mahanthesh and Thriveni [58], Shafiq et al. [59], Vahedi et al. [60], and Abdelmalek et al. [61]).

Hence, our main objective was to generate all available numerical solutions from the present model and conduct the statistical data analysis using response surface methodology. For the numerical solutions, the reduced system of linear equations was solved using the bvp4c solver. The selected data for the ANOVA were selected based on the central composite design in RSM. We believe that no similar work is being considered, which supports the novelty and significance of this work. From the ANOVA, the fitted model for the responses (skin friction coefficient and heat transfer rate) can be generated based on the physical factors (suction, wedge parameter, and volumetric concentration of hybrid nanoparticles). These equations can be used for practical and future applications regarding the mixed convection (opposing) flow subject to a permeable and vertical wedge.

## 2. Mathematical Formulation

**Figure 1:**
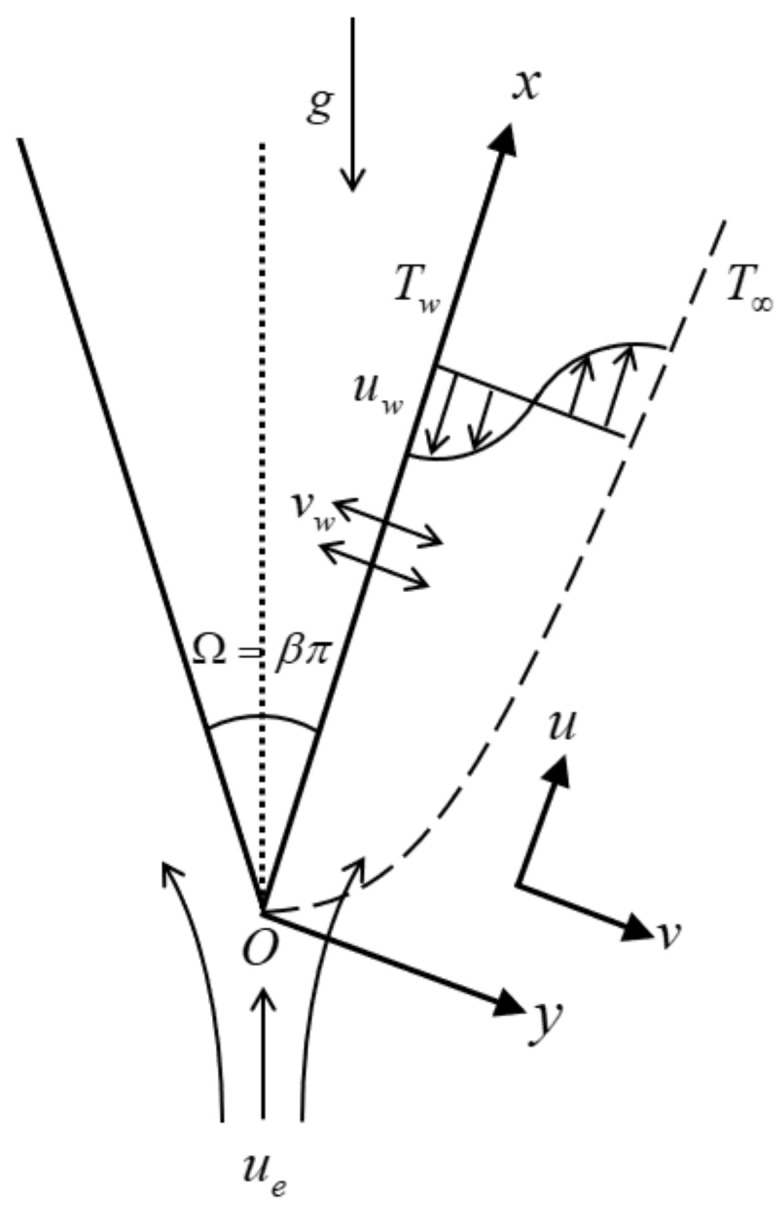
The physical model.

Consider a mixed convection and steady flow of a water-based hybrid nanofluid with copper–alumina (Cu-Al_2_O_3_) nanoparticles towards a permeable wedge. The free stream flow with velocity $u_{e}\left(x\right)=ax^{m}$ is assumed to move over the static wedge as portrayed in Figure 1, where a>0 is a constant while $m=\beta /\left(2-\beta \right)$ is a positive constant related to the angle of the wedge, and the chosen m must be within the interval of 0≤m≤1. Further, β is the Hartree pressure gradient and the total angle of the wedge (see Figure 1) is denoted as Ω=βπ (Waini et al. [48], Rosca et al. [62]). Other presumptions for this physical model are:

- The variable wall temperature is $T_{w}\left(x\right)=T_{\infty }+T_{0}{\left(x/L\right)}^{2m-1}$ where L is a characteristic length of the wedge, $T_{w}>T_{\infty }\;\left(T_{0}>0\right)$ corresponds to an assisting flow (heated wedge), while $T_{w}<T_{\infty }\;\left(T_{0}<0\right)$ denotes an opposing flow (cooled wedge);
- The far-field temperature $T_{\infty }$ is fixed (constant);
- Both nanoparticles and base fluid are in a thermal equilibrium state;
- The model excludes the effect of sedimentation/aggregation since the hybrid nanofluid is in a stable synthesis.

The governing flow and energy equations are [48,62]

(1) $$\frac{\partial u\;}{\partial x}+\frac{\partial v}{\partial y}=0,$$

(2) $$u\frac{\partial u}{\partial x}+v\frac{\partial u}{\partial y}=u_{e}\frac{du_{e}}{dx}+\frac{\mu_{hnf}}{\rho_{hnf}}\frac{\partial^{2}u}{\partial y^{2}}+{\left(\beta_{T}\right)}_{hnf}g\left(T-T_{\infty }\right)\cos\alpha ,\;$$

(3) $$\;u\frac{\partial T}{\partial x}+v\frac{\partial T}{\partial y}=\frac{k_{hnf}}{{\left(\rho C_{p}\right)}_{hnf}}\frac{\partial^{2}T}{\partial y^{2}},$$

with the boundary conditions

(4) $$\left.\begin{array}{l} u=0,v=v_{w}\left(x\right),T=T_{w}\left(x\right),\;\mathrm{at}\;y=0\\ u\to u_{e}\left(x\right),T\to T_{\infty },\;\mathrm{as}\;y\to \infty . \end{array}\right\}$$

Here $u\left(x-\mathrm{direction}\right)$ and $v\left(y-\mathrm{direction}\right)$ are the hybrid nanofluid velocities, $v_{w}$ is the mass velocity, T is the temperature of the working fluid, α=Ω/2 is the respective angle for the model and g is the gravitational acceleration [62]. The following similarity variables are introduced which complies Equation (1),

(5) $$\left.\begin{array}{l} u=ax^{m}f^{\prime }\left(\eta \right),v=-\sqrt{\frac{\left(m+1\right)a\nu_{f}}{2}}x^{\left(\frac{m-1}{2}\right)}\left[f\left(\eta \right)+\frac{m-1}{m+1}\eta f^{\prime }\left(\eta \right)\right],\theta \left(\eta \right)=\frac{T-T_{\infty }}{T_{w}-T_{\infty }},\\ \eta =y\sqrt{\frac{a\left(m+1\right)}{2\nu_{f}}}x^{\left(\frac{m-1}{2}\right)}. \end{array}\right\}$$

Hence, the respective surface mass flux velocity is

(6) $$v_{w}=-\sqrt{\frac{\left(m+1\right)a\nu_{f}}{2}}x^{\left(\frac{m-1}{2}\right)}S,$$

where *S*  represents the fluid suction or removal/injection. Further, by substituting Equation (5) into Equations (2)–(4), the following ODEs with the reduced BCs are obtained

(7) $$\left(\frac{\mu_{hnf}/\mu_{f}}{\rho_{hnf}/\rho_{f}}\right)f^{\prime \prime \prime }+ff^{\prime \prime }+\frac{2m}{m+1}\left(1-{f^{\prime }}^{2}\right)+\left(\frac{{\left(\rho \beta_{T}\right)}_{hnf}/{\left(\rho \beta_{T}\right)}_{f}}{\rho_{hnf}/\rho_{f}}\right)\frac{2}{m+1}\lambda \cos\alpha \theta =0,$$

(8) $$\frac{1}{\mathrm{Pr}}\frac{k_{hnf}/k_{f}}{{\left(\rho C_{p}\right)}_{hnf}/{\left(\rho C_{p}\right)}_{f}}\theta^{\prime \prime }+f\theta^{\prime }-\left(2m-1\right)f^{\prime }\theta =0,$$

(9) $$\left(\frac{\mu_{hnf}/\mu_{f}}{\rho_{hnf}/\rho_{f}}\right)f^{\prime \prime \prime }+ff^{\prime \prime }+\frac{2m}{m+1}\left(1-{f^{\prime }}^{2}\right)+\left(\frac{{\left(\rho \beta_{T}\right)}_{hnf}/{\left(\rho \beta_{T}\right)}_{f}}{\rho_{hnf}/\rho_{f}}\right)\frac{2}{m+1}\lambda \cos\alpha \theta =0,$$

where $\mathrm{Pr}={\left(C_{p}\mu \right)}_{f}/k_{f}$ (Prandtl number), λ=Gr/Rex2 (mixed convection parameter), $Gr=g{\left(\beta_{T}\right)}_{f}\left(T_{w}\left(x\right)-T_{\infty }\right)x^{3}/\nu_{f}{}^{2}$ (local Grashof number) and Rex=xuex/νf (local Reynolds number). Further information for the mixed convection parameter is λ<0, λ=0 and λ>0 stand for an opposing, pure forced and assisting flows, respectively. 

Following Takabi and Salehi [6], the correlations of hybrid nanofluid properties that were experimentally validated are shown in Table 1. These correlations are also used in many numerical studies regarding boundary layer flow. The exact properties of the pure water, Al_2_O_3,_ and Cu nanoparticles for the computational analysis are listed in Table 2 [63,64]. A copper-water nanofluid model is obtained by setting $\phi_{{\mathrm{Al}}_{2}O_{3}}=\phi_{1}=0\%$ and alumina-water nanofluid model when $\phi_{\mathrm{Cu}}=\phi_{2}=0\%$. Furthermore, a viscous fluid model is presentable if $\phi_{1}=\phi_{2}=0\%.$

The definition of the skin friction coefficient and local Nusselt number is

(10) $$C_{f}=\frac{\tau_{w}}{\rho_{f}u_{e}^{2}},\;Nu_{x}=\frac{xq_{w}}{k_{f}\left(T_{w}\left(x\right)-T_{\infty }\right)},$$

where *τ_w_* and *q_w_* are the wall shear stress and heat flux, respectively, defined as

(11) $$\tau_{w}=\mu_{hnf}{\left(\frac{\partial u}{\partial y}\right)}_{y=0},\;q_{w}=-k_{hnf}{\left(\frac{\partial T}{\partial y}\right)}_{y=0}.$$

Using Equations (5), (9) and (10),

(12) Rex1/2Cf=μhnfμfm+12f″0, Rex−1/2Nux=−khnfkfm+12θ′0.

## 3. Results and Discussion

**Figure 2:**
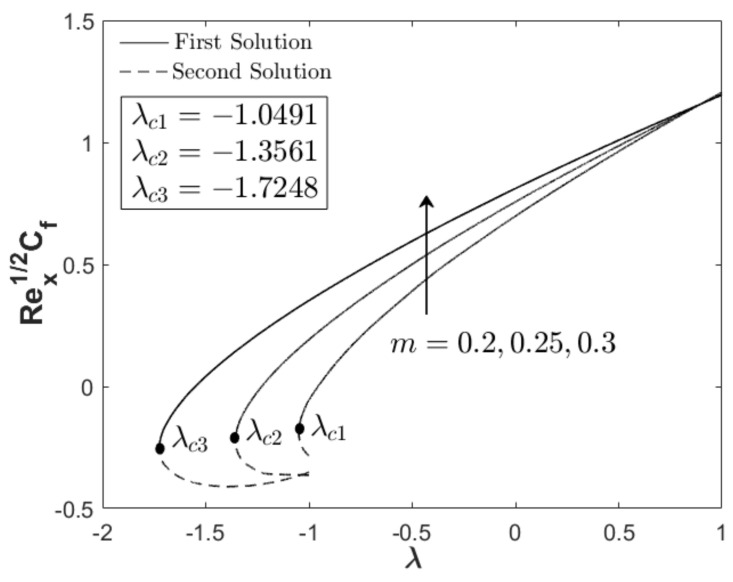
Rex1/2Cf towards λ when S=0.05, $\phi_{hnf}=0.02$, and different m.

**Figure 3:**
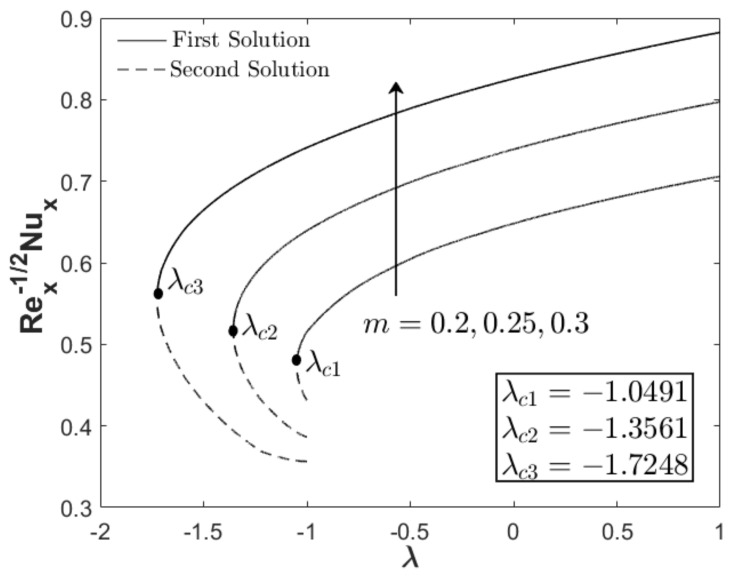
Rex−1/2Nux towards λ when S=0.05, $\phi_{hnf}=0.02$, and different m.

**Figure 4:**
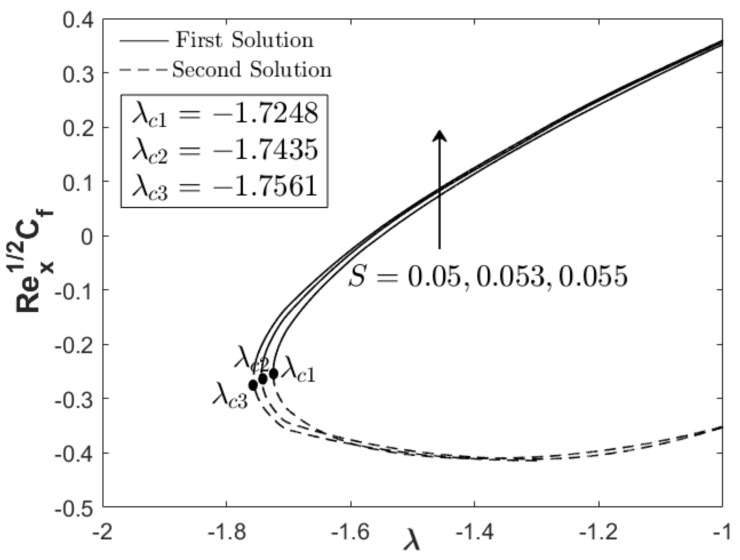
Rex1/2Cf towards λ when m=0.3, $\phi_{hnf}=0.02$, and variety S.

**Figure 5:**
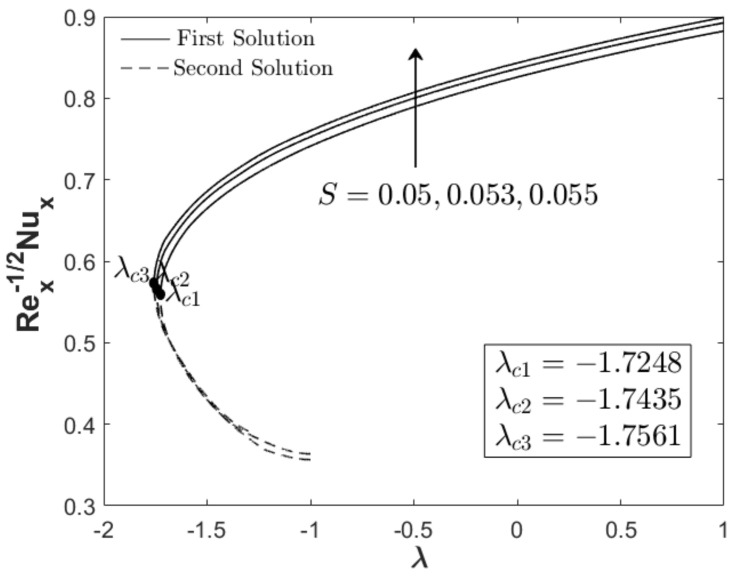
Rex−1/2Nux towards λ when m=0.3, $\phi_{hnf}=0.02$, and variety S.

**Figure 6:**
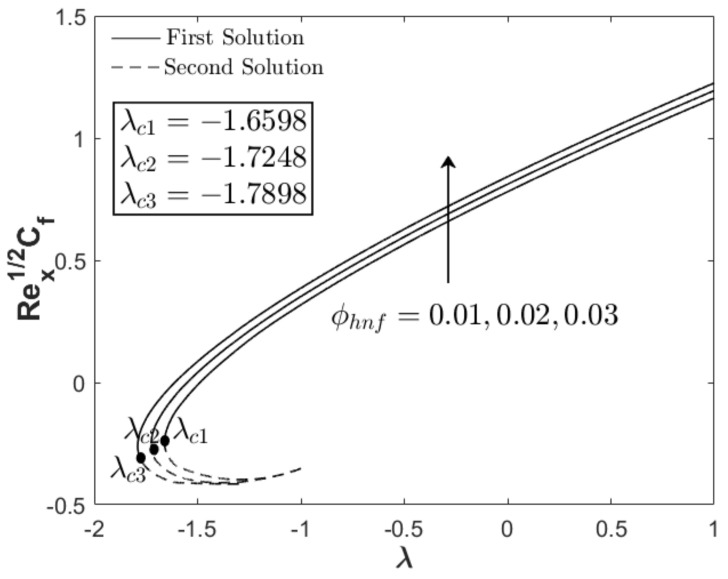
Rex1/2Cf towards λ when m=0.3, S=0.05, and different $\phi_{hnf}.$

**Figure 7:**
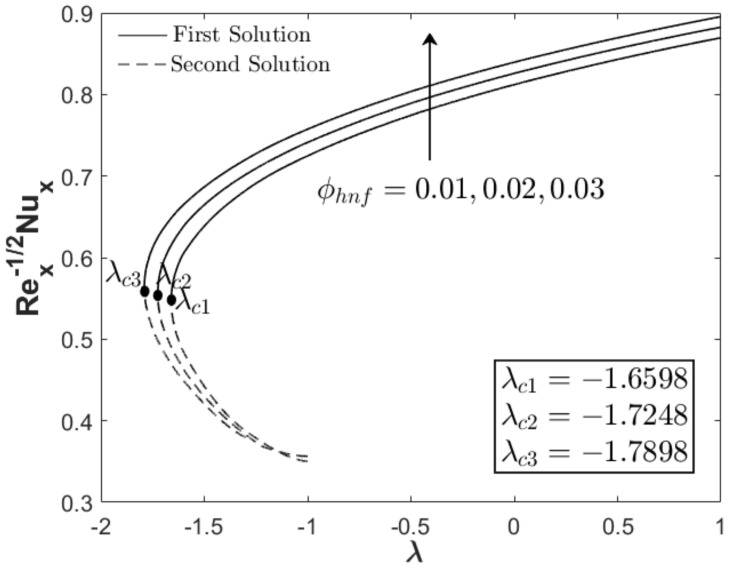
Rex−1/2Nux towards λ when m=0.3, S=0.05, and different $\phi_{hnf}.$

In this section, the results are discussed based on the numerical solutions of Equations (7)–(9) through the bvp4c application in the Matlab software. The thermal and flow performances of Al_2_O_3_-Cu/water hybrid nanofluid are observed and computed for three regions: when λ>0 (assisting flow solution), λ<0 (opposing flow solution) and λ=0 (pure force convection). For that reason, the effect wedge parameter m, suction S and concentration of the hybrid nanoparticles $\phi_{hnf}$ are numerically studied on the skin friction coefficient and thermal rate as displayed in Figure 2, Figure 3, Figure 4, Figure 5, Figure 6 and Figure 7. The numerical solutions and appearance of dual solutions are observed within this interval 0.2<m≤0.3, 0.05<S≤0.055, $0\%\leq \phi_{hnf}\leq 2\%$ and $\lambda_{c}<\lambda \leq 1$. The Prandtl number $\left(\mathrm{Pr}=6.2\right)$ is used which represents water as the carrier fluid. For the computational analysis, $\alpha =\frac{\beta \pi }{2}$ in Equation (7) is modified in term of m such that $\alpha =\frac{m}{m+1}\pi$ (angle in radians). For the model’s accurateness and validity, few solutions are validated by comparing them with existing literatures as presented in Table 3. Further, the observation of critical value $\lambda_{c}$ is necessary to find the final point of laminar flow separation. Usually, the critical value appears in the opposing flow region and beyond this value, no solution exists. Table 4 summarizes the critical values from Figure 2, Figure 3, Figure 4, Figure 5, Figure 6 and Figure 7 when different physical factors are considered. The expansion of the critical values is seen with the increment of m, S and $\phi_{hnf}$ which reveals that all these physical parameters are beneficial in the deceleration of the boundary layer separation. 

In addition to the projection of the critical values, Figure 2, Figure 3, Figure 4, Figure 5, Figure 6 and Figure 7 also exhibited the impact of the parameters on the flow behavior and thermal progress. In Figure 2 and Figure 3, the addition of wedge parameter m=0.2,0.25,0.3, which corresponds to the angle of the wedge α=30°,36°,41.5° (in degree), enhances both skin friction Rex1/2Cf and thermal rate Rex−1/2Nux. Moreover, both Rex1/2Cf and Rex−1/2Nux increase as the mixed convection parameter λ→1. Theoretically, the positive λ shows an assisting flow behavior that induces and aids fluid movement, including the active process of heat removal. Figure 4 and Figure 5 show the augmentation of Rex1/2Cf and Rex−1/2Nux with the increment of the suction parameter. However, the skin friction distribution was only slightly affected as compared to the heat transfer progress. As previously discussed, the limitation of suction magnitude was based on the observation of dual solutions. There is no restriction if the researchers use a higher magnitude of suction. The impact of volumetric concentration on the hybrid nanoparticles is presented in Figure 6 and Figure 7. It is worth mentioning that we considered an equal concentration of Cu and Al_2_O_3_ nanoparticles such that $\phi_{hnf}=0.01,0.02,0.03$ corresponds to $\phi_{1}=\phi_{2}=0.005,0.01,0.015$. The results show that both skin friction coefficient and thermal rate enhance with the increment of $\phi_{hnf}$.

## 4. Response Surface Methodology

**Figure 8:**
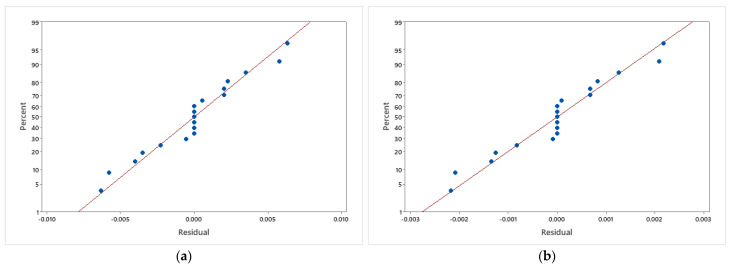
The residual normal plot for (**a**) Rex1/2Cf and (**b**) Rex−1/2Nux.

**Figure 9:**
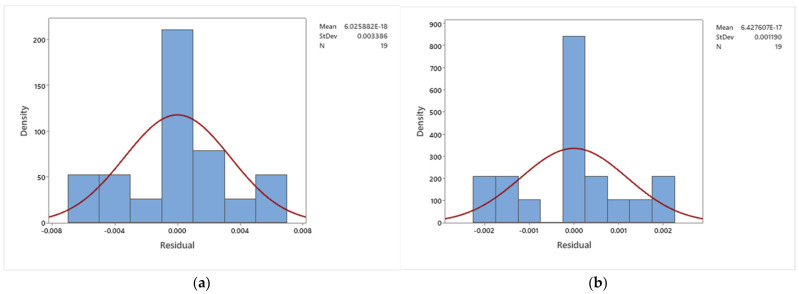
The distribution of residual from (**a**) Rex1/2Cf and (**b**) Rex−1/2Nux.

The experimental design for the particular set of data in the boundary layer flow problem is also important, where the researchers can estimate which parameters (factors) are influential or beneficial in optimizing the responses (skin friction coefficient/thermal rate). There are many types of experimental design available such as factorial design and response surface methodology through central composite design or Box–Behnken design. From the numerical interpretation, the suction, volumetric nanoparticles concentration, and wedge parameter affect and enhance both Rex1/2Cf and Rex−1/2Nux; however, from the statistical data analysis, the most significant factor contributing to the enhancement of responses can be predicted. Table 4 displays the wedge parameter, volumetric concentration of the hybrid nanoparticles, and suction parameter as the factors, and they are symbolized as A, B, and C, respectively. The level is referred to the magnitude of each factor and is categorized as low, medium, and high. As previously stated, the controlling parameters are used within the range of 0.2<m≤0.3, $0.01\leq \phi_{hnf}\leq 0.03$ and 0.05<S≤0.055. The division of low, medium, and high magnitudes of the parameters is also clearly stated in Table 5. The total runs for three factors $\left(k=3\right)$ with 5 centre points $\left(C=5\right)$ are based on this formula $R=2^{k}+2k+C$ where $2^{k}$ is the factorial points, 2k is the axial points, and C is the center points [57]. Table 6 shows the response surface methodology using a central composite design with 19 total runs when λ=−1 and Pr=6.2. By using the data in Table 6, the correlations between the factors $\left(m,\phi_{hnf},S\right)$ and responses Rex1/2Cf,Rex−1/2Nux can be defined by this general response surface Equation (13)

(13) $$y=r_{0}+r_{A}A+r_{B}B+r_{C}C+r_{A^{2}}A^{2}+r_{B^{2}}B^{2}+r_{C^{2}}C^{2}+r_{AB}AB+r_{CA}CA+r_{BC}BC+\varepsilon ,$$

where $r_{0}$ is an intercept, $r_{A},r_{B},r_{C}$ is the linear effects, $r_{A^{2}},r_{B^{2}},r_{C^{2}}$ is the quadratic effects and $r_{AB},r_{CA},r_{BC}$ is the interaction effects. Two response surface equations were considered for the two responses. The execution of data analysis was further conducted using analysis of variance (ANOVA) through the fit general linear model in statistical analysis Minitab software. The results are presented in Table 5, Table 6, Table 7, Table 8 and Table 9 and Figure 8 and Figure 9.

In the interest of producing a good model and well-fitted to the response-surface component, three main indicators need to be considered, which are the *p*-values (*p*-value < 0.001) from the analysis of variance (ANOVA) table, the value of adjusted R square (R-sq), and standard residual plot. Table 7 presents the ANOVA table to analyse the effect considered parameters such as wedge parameter (A), suction parameter (B), and volumetric concentration of nanoparticles (C) to the model of response-surface component for the skin friction coefficient, Rex1/2Cf, and heat transfer rate, Rex−1/2Nux. Based on the result of the *p*-values, it is apparent that the wedge parameter (A) and volumetric concentration of nanoparticles (C) have a significant impact on all two responses, Rex1/2Cf and Rex−1/2Nux. However, the model’s suction parameter (B) effect is only significant for. In addition, the symbol * in Table 7 shows that the value is too small. Model summary for Rex1/2Cf and Rex−1/2Nux, which include adjusted R-sq, is presented in Table 8.

This value is represented by how much the models explain the variation in response used. It was obtained that the value of adjusted R-sq for Rex1/2Cf and Rex−1/2Nux is 99.89% and 99.95%, respectively. Based on this result, it was shown that all models explain a very high percentage of the total variation in respective responses. The residual normal plot for both fitted models, Rex1/2Cf and Rex−1/2Nux, is presented in Figure 8. This result is used to evaluate the goodness of fit to the models of the response-surface component. A good model that accurately represents the relationship between behavioral input parameters and response reveals a one-to-one correlation between theoretical and observed quantiles. It was found that for both models, there is almost a one-to-one correlation between theoretical and observed quintiles. The distribution of residual from both fitted models Rex1/2Cf and Rex−1/2Nux is presented in Figure 9. It is shown that both fitted models’ residuals are normally distributed. Therefore, both models are well-fitted.

Table 9 presents the fitted model terms for the skin friction coefficient, Rex1/2Cf, and heat transfer rate, Rex−1/2Nux, to analyze the significant input parameters together with t-value and *p*-value with a 95% confidence interval. It was found that the wedge parameter (A) and volumetric concentration of nanoparticles (C) are significant terms affecting Rex1/2Cf, with a *p*-value < 0.001. Whereas the wedge parameter (A), suction parameter (B), and volumetric concentration of nanoparticles (C) are significantly (*p*-value < 0.001) affecting Rex−1/2Nux. Therefore, the corresponding fitted models for Rex1/2Cf and heat transfer rate by considering the three effects can be expressed as

(14) $$\begin{array}{rr} y_{\mathrm{skin}\;\mathrm{friction}}= & 0.19818+0.19995A+0.00663B+0.04001C-0.00428\mathrm{AB}\\ & -0.01084\mathrm{AC}-0.00024\mathrm{BC}-{0.04114A}^{2}-{0.00019B}^{2}-{0.00131C}^{2}, \end{array}$$

(15) $$\begin{array}{rr} y_{\mathrm{heat}\;\mathrm{transfer}}= & 0.651294+0.111817A+0.010414B+0.017393C-0.001419\mathrm{AB}\\ & -0.002866\mathrm{AC}-0.000153\mathrm{BC}-0.010382A^{2}-0.001627B^{2}-0.000664C^{2}. \end{array}$$

## 5. Conclusions

The flow behavior and thermal properties of Cu-Al_2_O_3_/water with mixed convection in the context of a permeable and vertical wedge were addressed and discussed in detail. The similarity transformation was used to simplify and reduce the partial differential equations into a set of ordinary (similarity) differential equations. The Matlab software, with its capable bvp4c function, was utilized to numerically compute the steady similarity solutions. The numerical solutions were then presented in the graphs of skin friction coefficient and heat transfer rate for various wedge parameters, the volumetric concentration of nanoparticles, and suction parameters. Moreover, for the statistical evaluation, the response surface methodology was used to gather the data and then analyzed using the analysis of variance (ANOVA) through the fit general linear model in the Minitab software. The following is the summary of the findings:

- The steady flow problem was solved for three cases: assisting flow, opposing flow, and pure force convective flow. The dual solutions were observable only in the opposing flow region when appropriate parameters were used;
- From the numerical evaluation, the addition of the wedge parameter, the volumetric concentration of nanoparticles, and the suction parameter contribute to the expansion of the critical value, which implies the delay in boundary layer separation. Furthermore, the skin friction coefficient and heat transfer process in the opposing flow region were also raised by these controlling parameters;
- From the statistical evaluation, the two responses (heat transfer rate and skin friction coefficient) were significantly affected by the wedge and volumetric concentration of nanoparticles factors. However, the effect of the suction parameter is only relevant for the heat transfer rate and not for the skin friction coefficient;
- Nonetheless, all models account for a significant part of the total variation in the responses. Moreover, the residuals of both fitted models were also demonstrated to be normally distributed and well-fitted.

## Figures and Tables

**Table 1:** Correlations of hybrid nanofluids.

Properties	Correlations
Thermal conductivity	$k_{hnf}=\left[\frac{\left(\frac{\phi_{1}k_{1}+\phi_{2}k_{2}}{\phi_{hnf}}\right)-2\phi_{hnf}k_{f}+2\left(\phi_{1}k_{1}+\phi_{2}k_{2}\right)+2k_{f}}{\left(\frac{\phi_{1}k_{1}+\phi_{2}k_{2}}{\phi_{hnf}}\right)+\phi_{hnf}k_{f}-\left(\phi_{1}k_{1}+\phi_{2}k_{2}\right)+2k_{f}}\right]k_{f}$
Thermal expansion	${\left(\rho \beta_{T}\right)}_{hnf}=\phi_{1}{\left(\rho \beta_{T}\right)}_{s1}+\phi_{2}{\left(\rho \beta_{T}\right)}_{s2}+\left(1-\phi_{hnf}\right){\left(\rho \beta_{T}\right)}_{f}$
Heat capacity	${\left(\rho C_{p}\right)}_{hnf}=\phi_{1}{\left(\rho C_{p}\right)}_{s1}+\phi_{2}{\left(\rho C_{p}\right)}_{s2}+\left(1-\phi_{hnf}\right){\left(\rho C_{p}\right)}_{f}$
Density	$\rho_{hnf}=\phi_{1}\rho_{s1}+\phi_{2}\rho_{s2}+\left(1-\phi_{hnf}\right)\rho_{f}$
Dynamic viscosity	$\mu_{hnf}=\frac{\mu_{f}}{{\left(1-\phi_{hnf}\right)}^{2.5}};\phi_{hnf}=\phi_{1}+\phi_{2}$

**Table 2:** Thermophysical properties for H_2_O, Al_2_O_3_, and Cu.

Properties	Water	Copper	Alumina
ρ $\left(\mathrm{kg}/m^{3}\right)$	997.1	8933	3970
$C_{p}$ $\left(J/\mathrm{kgK}\right)$	4179	385	765
k $\left(W/\mathrm{mK}\right)$	0.6130	400	40
$\beta_{T}$ $\left(S/m\right)$	$21\times 10^{-5}$	$1.67\times 10^{-5}$	$0.85\times 10^{-5}$
Prandtl number, $\left(\mathrm{Pr}\right)$	6.2	-	-

**Table 3:** Comparison of Rex1/2Cf with different value of $\phi_{2}$ when λ=S=0.

$\phi_{2}$	Present	Waini et al. [28]	Waini et al. [48]
0	1.2968902	1.296890	1.296890
0.25	1.5538496	1.553850	1.553850

**Table 4:** Summary of the critical values with different physical parameters.

m	S	$\phi_{hnf}$	$\lambda_{c}$
0.2	0.05	0.02	−1.0491
0.25			−1.3561
0.3			−1.7248
	0.053		−1.7435
	0.055		−1.7561
	0.05	0.01	−1.6598
		0.03	−1.7898

**Table 5:** Experimental design for the factors and their levels.

Factor	Symbol	Level		
		Low (−1)	Medium (0)	High (1)
m	A	0.2	0.25	0.3
S	B	0.05	0.053	0.055
$\phi_{hnf}$	C	0.01	0.02	0.03

**Table 6:** Response surface methodology with central composite design for the factors and responses.

Runs	Real Values			Coded Values			Responses	
	m	S	$\phi_{hnf}$	A	B	C	Skin Friction Coefficient	Heat Transfer Rate
1	0.2	0.053	0.03	−1	0	1	0.008650682	0.549364184
2	0.2	0.053	0.01	−1	0	−1	−0.100846497	0.506083442
3	0.25	0.053	0.02	0	0	0	0.198181909	0.651293721
4	0.3	0.05	0.02	1	−1	0	0.352159148	0.741277394
5	0.25	0.053	0.02	0	0	0	0.198181909	0.651293721
6	0.3	0.053	0.03	1	0	1	0.390631245	0.768679201
7	0.25	0.053	0.02	0	0	0	0.198181909	0.651293721
8	0.25	0.05	0.01	0	−1	−1	0.156111888	0.623220285
9	0.25	0.055	0.01	0	1	−1	0.167034194	0.643437992
10	0.3	0.053	0.01	1	0	−1	0.322494736	0.736190043
11	0.25	0.053	0.02	0	0	0	0.198181909	0.651293721
12	0.25	0.05	0.03	0	−1	1	0.226804209	0.65487521
13	0.25	0.053	0.02	0	0	0	0.198181909	0.651293721
14	0.25	0.055	0.03	0	1	1	0.236762452	0.674480548
15	0.3	0.055	0.02	1	1	0	0.35968123	0.760184189
16	0.2	0.05	0.02	−1	−1	0	−0.054533056	0.515546913
17	0.25	0.053	0.02	0	0	0	0.198181909	0.651293721
18	0.2	0.055	0.02	−1	1	0	−0.029896804	0.540129695
19	0.2	0.053	0.03	−1	0	1	0.008650682	0.549364184

**Table 7:** Analysis of variance (ANOVA) for the responses.

Source	DF	Seq SS	Contribution	Adj SS	Adj MS	F-Value	*p*-Value
Rex1/2Cf							
A	1	0.347418	94.01%	0.344435	0.344435	15024.57	0.000
B	1	0.000352	0.10%	0.000352	0.000352	15.34	0.004
C	1	0.013317	3.60%	0.013790	0.013790	601.55	0.000
A*B	1	0.000073	0.02%	0.000073	0.000073	3.19	0.108
A*C	1	0.000238	0.06%	0.000549	0.000549	23.93	0.001
B*C	1	0.000000	0.00%	0.000000	0.000000	0.01	0.922
A*A	1	0.007931	2.15%	0.007683	0.007683	335.16	0.000
B*B	1	0.000000	0.00%	0.000000	0.000000	0.01	0.933
C*C	1	0.000008	0.00%	0.000008	0.000008	0.34	0.574
Error	9	0.000206	0.06%	0.000206	0.000023		
Lack-of-Fit	3	0.000206	0.06%	0.000206	0.000069	*	*
Pure Error	6	0.000000	0.00%	0.000000	0.000000		
Total	18	0.369543	100.00%				
Rex−1/2Nux							
A	1	0.108745	96.40%	0.107718	0.107718	38033.61	0.000
B	1	0.000868	0.77%	0.000868	0.000868	306.35	0.000
C	1	0.002602	2.31%	0.002606	0.002606	920.21	0.000
A*B	1	0.000008	0.01%	0.000008	0.000008	2.84	0.126
A*C	1	0.000019	0.02%	0.000038	0.000038	13.54	0.005
B*C	1	0.000000	0.00%	0.000000	0.000000	0.03	0.860
A*A	1	0.000519	0.46%	0.000489	0.000489	172.82	0.000
B*B	1	0.000013	0.01%	0.000012	0.000012	4.24	0.070
C*C	1	0.000002	0.00%	0.000002	0.000002	0.71	0.423
Error	9	0.000025	0.02%	0.000025	0.000003		
Lack-of-Fit	3	0.000025	0.02%	0.000025	0.000008	*	*
Pure Error	6	0.000000	0.00%	0.000000	0.000000		
Total	18	0.112801	100.00%	0.112801			

The symbol * shows that the value is too small.

**Table 8:** Model summary for the responses.

S	R-sq	R-sq (adj)	R-sq (pred)
Rex1/2Cf			
0.0047880	99.94%	99.89%	99.19%
Rex−1/2Nux			
0.0016829	99.98%	99.95%	99.67%

**Table 9:** Fitted model terms for the responses.

Term	Coef	SE Coef	95% CI	T-Value	*p*-Value	VIF
Rex1/2Cf						
Constant	0.19818	0.00195	(0.19376, 0.20260)	101.39	0.000	
A	0.19995	0.00163	(0.19626, 0.20364)	122.57	0.000	1.04
B	0.00663	0.00169	(0.00280, 0.01046)	3.92	0.004	1.00
C	0.04001	0.00163	(0.03632, 0.04370)	24.53	0.000	1.04
A*B	−0.00428	0.00239	(−0.00969, 0.00114)	−1.79	0.108	1.00
A*C	−0.01084	0.00222	(−0.01586, −0.00583)	−4.89	0.001	1.06
B*C	−0.00024	0.00239	(−0.00566, 0.00517)	−0.10	0.922	1.00
A*A	−0.04114	0.00225	(−0.04622, −0.03605)	−18.31	0.000	1.04
B*B	−0.00019	0.00225	(−0.00528, 0.00489)	−0.09	0.933	1.02
C*C	−0.00131	0.00225	(−0.00639, 0.00377)	−0.58	0.574	1.04
Rex−1/2Nux						
Constant	0.651294	0.000687	(0.649740, 0.652848)	947.97	0.000	
A	0.111817	0.000573	(0.110520, 0.113114)	195.02	0.000	1.04
B	0.010414	0.000595	(0.009068, 0.011760)	17.50	0.000	1.00
C	0.017393	0.000573	(0.016096, 0.018690)	30.33	0.000	1.04
A*B	−0.001419	0.000841	(−0.003322, 0.000485)	−1.69	0.126	1.00
A*C	−0.002866	0.000779	(−0.004629, −0.001104)	−3.68	0.005	1.06
B*C	−0.000153	0.000841	(−0.002057, 0.001750)	−0.18	0.860	1.00
A*A	−0.010382	0.000790	(−0.012169, −0.008596)	−13.15	0.000	1.04
B*B	−0.001627	0.000790	(−0.003413, 0.000160)	−2.06	0.070	1.02
C*C	−0.000664	0.000790	(−0.002450, 0.001123)	−0.84	0.423	1.04

## Data Availability

The data presented in this study are available on request from the corresponding author.
